# Constricting Life Space and Likelihood of Neurodegenerative Disease in Community-Dwelling Older Men

**DOI:** 10.1001/jamanetworkopen.2023.42670

**Published:** 2023-11-09

**Authors:** Meredith A. Bock, Tina Hoang, Peggy Cawthon, Dawn C. Mackey, Sheena Patel, Teresa A. Hillier, Kristine Yaffe

**Affiliations:** 1 Department of Neurology, University of California, San Francisco; 2 California Pacific Medical Center Research Institute, San Francisco; 3 Department of Epidemiology and Biostatistics, University of California, San Francisco; 4 San Francisco Veterans Affairs Medical Center, San Francisco, California; 5 Department of Biomedical Physiology and Kinesiology, Simon Fraser University, Burnaby, Canada; 6 Centre for Aging SMART, Vancouver Coastal Health Research Institute and the University of British Columbia, Vancouver, Canada; 7 Kaiser Permanente Center for Health Research, Portland, Oregon; 8 Department of Psychiatry, University of California, San Francisco

## Abstract

**Importance:**

Life space is a measure of the frequency, range, and independence of movement through the environment. There is increasing interest in life space as a holistic measure of function in older adults, but the association between change in life space and incident neurodegenerative disease is unknown.

**Objective:**

To evaluate the association between change in life space and cognitive decline or incident neurodegenerative disease over 7 years among community-dwelling older men.

**Design, Setting, and Participants:**

In this cohort study, logistic regression analyses were used to examine the association of baseline and change in life space with change in cognition unadjusted and adjusted for demographics, cardiovascular risk factors, depression, gait speed, and physical activity. Mixed linear effects models were used to evaluate the association between change in life space and change in cognition. Men were recruited from 6 US sites to participate in a prospective, community-based cohort study of aging and followed-up from 2007 to 2014. Individuals with prevalent dementia or Parkinson disease (PD) at baseline were excluded. Data were analyzed from May 2022 to September 2023.

**Exposure:**

Life space, assessed using the University of Alabama at Birmingham Life Space Assessment and divided into tertiles.

**Main Outcomes and Measures:**

Participants completed the Modified Mini-Mental State (3MS) Test, and Trail-Making Test Part B at baseline and 7 years later. At follow-up, participants were asked about a new physician diagnosis of dementia and PD.

**Results:**

A total of 1684 men (mean [SD] age, 77.1 [4.2] years) were recruited and over 7 years of follow-up, 80 men (4.8%) developed dementia and 23 men (1.4%) developed PD. Mean (SD) life space score was 92.9 (18.7) points and mean (SD) change was −9.9 (22.3) points over follow up. In the adjusted model, each 1-SD decrement in life space was associated with increased odds of dementia (odds ratio [OR], 1.59; 95% CI, 1.28-1.98) but not PD (OR, 1.48; 95% CI, 0.97-2.25). For each 1-SD decrement in life space, men worsened by 20.6 (95% CI, 19.8-21.1) seconds in their Trails B score (*P* < .001) and declined by 1.2 (95% CI, 1.0-1.3) points in their 3MS score (*P* < .001) over 7 years.

**Conclusions and Relevance:**

In this study of 1684 men followed up over 7 years, change in life space was associated with faster cognitive decline and increased likelihood of neurodegenerative illness. Future studies should examine the role of clinician assessments or wearable electronics in tracking life space in older adults at risk of cognitive decline and neurodegenerative illness.

## Key Points

**Question:**

Is change in life space (the frequency, range, and independence of movement through the environment) associated with incident neurodegenerative disease or cognitive decline in community-dwelling older men?

**Findings:**

In this cohort study of 1684 men, a decline in life space by 1 SD of the population mean was associated with approximately 60% increased odds of incident dementia and decline in global cognition and executive function over 7 years.

**Meaning:**

These findings suggest that clinician assessments of life space or wearable electronics may help identify older adults at risk of cognitive decline and neurodegenerative disease.

## Teaser

This cohort study evaluates associations of change in life space, a measure of the frequency, range, and independence of movement through the environment, with cognitive decline or incident neurodegenerative disease over 7 years among community-dwelling older men.

## Introduction

Life space is a measure of the frequency, range, and independence of movement through the environment during daily life. There is increasing interest in life space as a more holistic measure of everyday function than typical self-reported measures of disability, which measure capacity for movement rather than actual enacted mobility.^1,2^ Restricted life space is associated with depression,^3^ frailty,^3^ mortality,^4,5,6^ and other adverse outcomes highly relevant to older adults. As more expansive life space relies on the application of a variety of functional skills, there is high interest in understanding whether restricted life space is an indicator of increased risk of cognitive decline and neurodegenerative disease.^7^

Prior studies on the association of life space with cognitive outcomes are primarily cross-sectional and have yielded mixed results.^1,3,8,9,10^ Preliminary studies have explored correlates of life space in diagnosed PD,^11,12^ but to our knowledge, none have evaluated life space as a risk factor. Furthermore, no study has evaluated change in life space and its association with neurodegeneration. Our goal was to evaluate whether change in life space is associated with a new diagnosis of dementia or PD or objectively measured cognitive decline in a community-based sample of older men.

## Methods

This cohort study was approved by institutional review boards at each site and the San Francisco coordinating center (California Pacific Medical Center and the University of California, San Francisco). All participants provided written informed consent. These analyses followed Strengthening the Reporting of Observational Studies in Epidemiology (STROBE) reporting guideline.

### Participants

The Osteoporotic Fractures in Men Study (MrOS) is a prospective cohort study of aging that enrolled 5994 men from 6 sites in the United States (Birmingham, Alabama; Minneapolis, Minnesota; Palo Alto, California; the Monogahela Valley, Pennsylvania; Portland, Oregon; and San Diego, California).^13^ Men were enrolled between March 2000 and April 2002 through mailings using community and clinician contact lists, newspaper advertisements, and presentations to older adults.^14^ All participants were aged 65 years or older and living independently in the community at baseline. They were invited to complete 12 evaluations over 17 years, and follow-up is ongoing.

Of 5994 participants enrolled in the MrOS study, 3892 had a life space assessment at year 7 (our study baseline, 2007). Of these participants, 2116 had a second life space assessment 7 years later (2014). We excluded 56 of these participants as they had probable dementia at baseline (defined as self-reported physician diagnosis of dementia, Modified Mini-Mental State Examination score <80, or use of dementia medication) or a self-reported physician diagnosis of PD. We excluded an additional 376 individuals who did not have cognitive data at both time points. Our final analytic cohort included 1684 individuals (eFigure 1 in Supplement 1).

### Life Space Measure

Participants reported life space using the University of Alabama at Birmingham Life Space Assessment (eFigure 2 in Supplement 1).^2^ This tool asks about an individual’s movement in the last 4 weeks to rooms in the home, outside the home (eg, porch, deck, patio, yard), in the neighborhood, out of the neighborhood, and out of town. Participants self-defined which area constituted their neighborhood or town. For each of the areas, the participant was asked about their frequency of travel to that location (less than once per week, 1-3 times per week, 4-6 times per week, or daily) and their level of independence (requiring assistance from another person, aids or equipment, or no assistance). The total life space score is the life space area multiplied by frequency and level of independence, yielding a score ranging from 0 (never leaving one’s bedroom) to 120 (regularly traveling out of town).

### Cognitive Measures

All participants completed 2 administered cognitive tests at the study baseline and 7 years later. The Modified Mini-Mental State Examination (3MS) was used to assess global cognition with questions on orientation, concentration, language, praxis, and immediate and delayed memory and is scored 0 (worst cognition) to 100 (best cognition).^15^ The Trails B test was used to measure executive function by asking participants to set shift by connecting alternating numbers and letters.^16^ Participants were timed in their completion of this task and allowed up to 300 seconds, with more time needed to complete indicating worse performance.

### Ascertainment of Alzheimer or PD

Study participants were asked about a physician diagnosis of dementia (phrased as “dementia or Alzheimer’s disease”) or PD at the study baseline and 7 years later. Incident dementia was defined as a new self-reported physician diagnosis of dementia. Incident PD was defined as a new self-reported physician diagnosis of PD.

### Other Variables

Participants completed questionnaires to report their age, race and ethnicity, educational level, living status (alone or with others), marital status, smoking status, and independence in their activities of daily living. Race and ethnicity were categorized as Asian, Black, Hispanic, White, or other (eg, includes American Indian, Native Hawaiian or Pacific Islander, or individuals who identified as multiple races). Multiple racial and ethnic groups were included in demographic features because previous work has indicated possible health disparities in dementia. Participants completed the Geriatric Depression scale, a well-validated assessment scored from 0 to 15, with a score greater than 6 coded as likely clinical depression.^17^ Participant height and weight were measured at baseline, which was used to calculate body mass index (calculated as weight in kilograms divided by height in meters squared). Cardiovascular risk factors (diabetes, stroke, and hypertension) were ascertained at the study baseline. Physical activity was evaluated with the Physical Activity Scale for the Elderly (PASE) score.^18^ As part of the Short Form–12-item,^19^ participants were asked how they would rate their health compared with other people their age, and we coded responses as a dichotomous variable with excellent or good self-rated health in one category and very poor, poor, or fair coded in the other category. Gait speed was measured as the fastest time in meters per second on a 6-m walking test.

### Statistical Analysis

Baseline participant characteristics were examined by tertiles of life space change. To examine the association between baseline life space and incident dementia and PD, we performed logistic regression analyses. We first analyzed the association of life space tertile with incident dementia and PD. We then analyzed the association of change in life space (as a continuous variable) with incident dementia and PD. To further evaluate for reverse causality, we performed sensitivity analyses excluding individuals with baseline restricted life space and baseline cognitive impairment. We also performed sensitivity analysis for the association between life space and incident PD, restricting our analysis only to those with incident PD as defined by self-reported physician-diagnosed PD and dopaminergic medication use.

To evaluate the association between change in life space and change in cognition over 7 years, we performed random-effects regression models. These models account for between-participant variation and within-participant correlations for repeated measures. Model coefficients were estimated using the restricted maximum likelihood method, and time was modeled as the age at the time of each visit and centered on mean age. All multivariable models were further adjusted for clinic site, race and ethnicity, education, living alone, diabetes, stroke, hypertension, depression, gait speed, and physical activity (PASE score). Outcomes were transformed for normality (squared for 3MS score and natural log for Trails B time) and were back transformed. Analyses used SAS statistical software version 9.4 (SAS Institute). *P* values were 2-tailed, and statistical significance was set at *P* < .05. Data were analyzed from May 2022 to September 2023.

## Results

### Sample Characteristics

A total of 1684 (mean [SD] age, 77.1 [4.2] years) were included in analysis. Demographic characteristics of the analytic cohort are summarized in Table 1. At our study baseline, the mean (SD) composite life space score was 92.9 (18.7) points. There were 509 participants (30.2%) in the most restricted life space tertile, 387 participants (23.0%) in the moderately restricted life space tertile, and 788 participants (46.8%) in the least restricted life space tertile. Participants with more restricted life space at baseline were significantly more likely to be older and more likely to report fewer years of education, lower physical activity, and greater rates of depression (Table 1).

**Table 1.** Baseline Characteristics of Men in the Analytic Cohort by Life Space Tertile

Characteristic	Baseline life space score, Patients, No. (%) (N = 1684)^a^			*P* value^b^
	Most restricted (n = 509)	Moderately restricted (n = 387)	Least restricted (n = 788)	
Age, mean (SD), y	77.8 (4.6)	77.0 (4.1)	76.7 (3.9)	<.001
Race and ethnicity				
Asian	23 (1.4)	5 (0.3)	22 (1.3)	<.001
Black	21 (1.3)	12 (0.7)	8 (0.5)	
Hispanic	17 (1.0)	2 (0.1)	15 (0.9)	
White	437 (26.0)	360 (21.4)	737 (43.8)	
Other^c^	11 (0.7)	8 (0.5)	6 (0.4)	
Education level				
<High school	27 (5.3)	11 (2.8)	18 (2.3)	.03
High school graduate	78 (15.3)	56 (14.5)	106 (13.5)	
College or graduate school	404 (79.4)	320 (82.7)	664 (84.3)	
Married	406 (79.8)	329 (85.0)	647 (82.1)	.13
Lives alone	91 (17.9)	46 (11.9)	115 (14.6)	.04
BMI, mean (SD)	27.3 (3.5)	27.3 (3.5)	27.2 (3.7)	.81
Walking speed, mean (SD), m/s	1.2 (0.3)	1.3 (0.2)	1.3 (0.2)	<.001
Physical activity score, mean (SD)	130.3 (64.1)	145.7 (61.1)	160.4 (68.2)	<.001
Season of life-space assessment				
Fall	104 (20.4)	84 (21.7)	163 (20.7)	.25
Spring	163 (32.0)	128 (33.1)	281 (35.7)	
Summer	148 (29.1)	94 (24.3)	221 (28.1)	
Winter	94 (18.5)	81 (20.9)	123 (15.6)	
Excellent or good self-rated health	448 (88.2)	359 (92.8)	743 (94.4)	<.001
Current smoker	20 (3.9)	12 (3.1)	16 (2.0)	.19
Any ADL limitation	93 (18.3)	43 (11.1)	74 (9.4)	<.001
Any IADL limitation	71 (14.0)	23 (5.9)	45 (5.7)	<.001
Diabetes	82 (16.1)	48 (12.4)	77 (9.8)	.003
Stroke	22 (4.3)	13 (3.4)	35 (4.4)	.67
Hypertension	273 (53.7)	199 (51.4)	374 (47.5)	.08
Depression, GDS ≥6	21 (4.1)	7 (1.8)	15 (1.9)	.03
3MS score, mean (SD)	93.8 (4.3)	94.8 (3.7)	95.0 (3.4)	<.001
Trails B score, mean (SD)	113.1 (52.5)	101.7 (42.2)	96.8 (42.2)	<.001

Abbreviations: 3MS, Modified Mini-Mental State; ADL, activities of daily living; BMI, body mass index (calculated as weight in kilograms divided by height in meters squared); GDS, Geriatric Depression Scale; IADL, instrumental activities of daily living.

^a^ The most restricted tertile is a life space score of 21 to 85; moderately restricted, 86 to 99; and least restricted, 100 to 120.

^b^ *P* values are determined by analysis of variance tests for continuous variables and χ^2^ tests for homogeneity for categorical variables.

^c^ Includes American Indian, Native Hawaiian or Pacific Islander, or individuals who identified as multiple races.

MrOS study participants excluded from our analysis because they had missing life space or cognitive assessments were significantly older (mean [SD] age, 81 [5.5]) than included participants (*P* < .001). Excluded participants also had lower baseline life space scores (mean [SD] score, 78.8 [26.1] points; *P* < .001) as well as worse cognitive performance on the 3MS (mean [SD] score, 89.8 [8.7] vs 94.6 [3.8]; *P* < .001) and Trails B (mean [SD] score, 146.6 [72.8] seconds vs 102.8 [46.0] seconds; *P* < .001) assessments compared with included participants.

At a follow-up a mean (SD) of 7.3 (0.5) years later, the mean (SD) composite life space score in the overall sample declined by 9.9 points to 83.0 (23.0) points. There were 996 participants (59.1%) who reported a decline in life space score over 7 years, 240 participants (14.3%) who reported no change, and 448 participants (26.6%) who reported an improvement. Over 7 years of follow-up, 80 individuals developed incident dementia and 23 individuals developed incident PD. In participants with a new diagnosis of dementia reported 7 years after the study baseline, the mean (SD) life space composite score declined by 20.2 points from 90.5 (20.6) points to 70.3 (26.3) points. In participants with a new diagnosis of PD reported 7 years after the study baseline, the mean (SD) life space composite score declined by 21.3 points, from 88.9 (24.5) points to 67.6 (30.1) points.

### Life Space and Incident Neurodegenerative Illness

Table 2 summarizes the logistic regression analyses evaluating the association between baseline life space tertile and change in life space with cognitive outcomes. There was no significant association of restricted life space with odds of incident dementia (most vs least restricted: odds ratio [OR], 1.40; 95% CI, 0.83-2.37) or PD (most vs least restricted: OR, 1.25; 95% CI, 0.42-3.72). However, a decline in life space by 1 SD or greater was associated with 59% increased odds of dementia in the unadjusted model (OR, 1.59; 95% CI, 1.28-1.98) and 57% increased odds of dementia in the fully adjusted model (OR, 1.57; 95% CI, 1.24-1.98). A decline in life space by 1 SD was also associated with 63% increased odds of PD (OR, 1.63; 95% CI, 1.11-2.41) in the unadjusted model. After adjusting for all covariates, decline in life space was not associated with increased odds of PD (OR, 1.48; 95% CI, 0.97-2.25). In a sensitivity analysis for the PD group, we restricted our outcome to only the 17 individuals who reported both a new diagnosis of PD and dopaminergic medication use. There was no statistically significant association in this small sample (eTable 1 in Supplement 1). In our sensitivity analysis excluding 87 individuals with baseline restricted life space, our results were not significantly changed (eTable 2 in Supplement 1). In our sensitivity analysis excluding 89 individuals with baseline cognitive impairment, our results were not significantly different, and there was no statistically significant association of change in life space and incident PD in the fully adjusted model (eTable 3 in Supplement 1).

**Table 2.** Association of Baseline Life Space and Change in Life Space With Incident Alzheimer Disease and Parkinson Disease Over 7 Years

Measure	Odds ratio (95% CI)			
	Incident dementia (n = 80)		Incident Parkinson disease (n = 23)	
	Model 1^a^	Model 2^b^	Model 1^a^	Model 2^b^
Life space tertile				
Least restricted	1 [Reference]	1 [Reference]	1 [Reference]	1 [Reference]
Moderately restricted	0.54 (0.27-1.09)	0.53 (0.26-1.10)	1.36 (0.48-3.86)	1.23 (0.43-3.51)
Most restricted	1.41 (0.87-2.28)	1.40 (0.83-2.37)	1.39 (0.53-3.62)	1.25 (0.42-3.72)
*P* value^c^	.22	.26	.49	.71
Life space change				
1-SD decrease in life space score^d^	1.59 (1.28-1.98)	1.57 (1.24-1.98)	1.63 (1.11-2.41)	1.48 (0.97-2.25)
*P* value	<.001	.001	.01	.07

^a^ Model 1 is unadjusted.

^b^ Model 2 is adjusted for age, clinic site, race and ethnicity, education, living alone, diabetes, stroke, hypertension, depression, gait speed, and physical activity.

^c^ *P* value is for the trend across life space tertiles.

^d^ An 1-SD change in life space is 21.9 points.

### Life Space and Cognitive Decline

We performed additional analyses to examine whether change in life space was associated with change in global cognition or executive function over the same time course (Table 3). For each 1-SD decline in life space, individuals worsened by a mean of 1.2 (95% CI, 1.0-1.3) points on 3MS score (*P* < .001) and 20.6 (95% CI, 19.8-21.1) seconds on Trails B evaluation (*P* < .001) in the fully adjusted model.

**Table 3.** Association of Change in Life Space Score With Change in 3MS and Trails B Score

Life space	Mean change in score			
	3MS (95% CI), points		Trails B (95% CI), s	
	Model 1^a^	Model 2^b^	Model 1^a^	Model 2^b^
Decrease in life space score, 1 SD^c^	−1.3 (1.1 to 1.4)	−1.2 (1.0 to 1.3)	23.3 (24.4 to 21.9)	20.6 (19.8 to 21.1)
*P* value	<.001	<.001	<.001	<.001

Abbreviation: 3MS, Modified Mini-Mental State.

^a^ Model 1 is unadjusted.

^b^ Model 2 is adjusted for age, clinic site, race and ethnicity, education, living alone, diabetes, stroke, hypertension, depression, gait speed, and physical activity.

^c^ An 1-SD change in life space is 21.9 points.

## Discussion

In this cohort study including a community-dwelling sample of older men, decline in life space was associated with an approximately 60% increased odds of dementia over 7 years as well as worsening global cognition and executive function. There was a smaller sample size of individuals with incident PD in our cohort, and there was no significant association between declining life space and odds of developing PD in our fully adjusted model. Older men with a new diagnosis of dementia or PD exhibited a decline in life space that was twice as fast compared with older men without those diagnoses. Our study provides novel evidence for change in life space as an early marker or manifestation associated with neurodegenerative disease.

Our findings suggest that restriction in a person’s movement through the environment in everyday life indicates an increased likelihood of developing neurodegenerative illness. Life space has been of particular interest as a possible marker associated with early cognitive decline, as it measures performed actions (what a person does do) rather than theoretical abilities (what a person physically can do). Because it is influenced by cognitive, psychosocial, physical, environmental, and financial factors, life space is a more comprehensive measure of function in everyday life than traditional measures of disability.^20^ Our findings on the association between change in life space and increased likelihood of incident dementia significantly expand on existing literature, which comprises cross-sectional studies with mixed results^1,3,8,9,10^ and few longitudinal studies showing restricted life space confers a moderately increased risk of incident AD^21^ or cognitive decline^21,22,23^ over short follow-up. Our study drew from a large, well-characterized cohort. We had access to repeated measures of the expanded life space measure as our exposure and to adjust for several important possible confounders with robust results.

To our knowledge, no prior studies have investigated life space and incident PD. There is 1 small cross-sectional study^12^ and 1 mixed methods study^11^ in diagnosed PD showing that factors associated with decreased life space may include perceived walking difficulties, loss of ability to drive, need of caregiving, decreased social engagement, and lower financial resources. This study did not find that change in life space was associated with a statistically significant likelihood of later PD diagnosis, but it was limited by small sample size and warrants further evaluation in larger cohorts. These findings can inform ongoing efforts to find measures that are more responsive to change in prodromal or early PD than current standard clinical assessments.^24^ In our sensitivity analysis including only men with PD ascertained by both physician diagnosis and dopaminergic medication use (17 of 23 individuals with PD), our results were not statistically significant. This could be due to a lesser change in life space for those being treated for their PD or decreased power from small sample sizes, so this association will need to be further explored in larger cohorts.

There are several possible explanations for the association between declining life space and an increased risk of neurodegenerative illness. As there is evidence of AD and PD pathology in the brain years before the clinical diagnosis,^25,26^ one hypothesis is that constricted life space results from prodromal neurodegeneration. There are changes in physical function in AD years before detectable cognitive impairment,^27^ which may affect mobility. However, worsening life space was associated with comparably increased odds of developing dementia even after adjusting for gait speed in our analyses. Other features of prodromal neurodegeneration, such as changes in mood^28,29,30^ and motivation,^31,32^ may also be contributing to decreasing life space mobility. Second, a decline in life space before or concurrent with a diagnosis of neurodegenerative disease could indicate the presence of early cognitive changes. We excluded individuals with probable dementia (self-reported diagnosis of dementia, use of dementia medications, or a score less than 80 on the 3MS) at our study baseline and did a sensitivity analysis excluding those with baseline cognitive impairment and baseline restricted life space, but this does not rule out preexisting cognitive change below the measurable threshold. Our finding that change in life space was associated with measurable decline in global cognition and executive function further supports a connection between changing life space and early, subtle cognitive decline. Our study adds to prior work suggesting an association between life space mobility and executive function in particular,^33,34^ likely due to the centrality of many executive functions in navigating a complex or unfamiliar environment. Third, there could be a bidirectional association because the environmental complexity and richness associated with a more expansive life space could protect against cognitive decline.^35,36^ Increased spatial mobility also enables more social engagement and leisure activities, which further support cognition.^37,38,39^ In this case, environmental complexity is a modifiable risk factor worth exploring, with 1 study demonstrating short-term efficacy of a home-based intervention to improve life-space mobility in older adults.^40^ Lastly, although there was little change in the magnitude of association between change in life space and a new diagnosis of dementia after adjustment for many possible confounders (including depression, gait speed, and vascular risk factors), the possibility of unmeasured confounding remains.

### Limitations

This study has some limitations, including the use of self-reported diagnosis for diagnosis of dementia and PD. Prior studies have shown high concordance between self-reported and clinically determined PD^41,42^ and that self-reported dementia diagnosis may underestimate actual prevalence.^43^ Individuals who developed cognitive impairment or dementia by the follow-up visit may not have accurately reported their life space and contributed to bias in an unknown direction. We also restricted our analyses to individuals who had life space and cognitive measurements at both time points. Because individuals with missing data had worse baseline life space scores and worse cognitive performance, our results are likely biased toward the null hypothesis. Our study measures change in life space over 7 years and concurrent cognitive decline or incident neurodegenerative diagnosis, so we cannot fully elucidate the directionality of the association. Additionally, our study was restricted to a primarily White cohort of community-dwelling men, and there is evidence of differences in life space by gender and race and ethnicity,^7,10^ so the associations found in this study may not be generalizable to other demographic groups. Our number of outcomes was relatively small, especially for the cohort of individuals who developed PD, and further research is needed in larger sample sizes.

## Conclusions

This cohort study found that change in life space is associated with increased risk of dementia as well as cognitive decline in older men. Change in life space over time maybe be one way that clinicians can identify those in depth and time intensive cognitive testing, monitor individuals at high risk of cognitive decline, and inform the development of risk scores aiming to identify individuals with prodromal neurodegeneration. This study can also inform ongoing efforts to collect data with wearable electronics in older adults,^44^ as changes within the same individual over time may have higher predictive value than baseline assessments.

## Notes

Supplement 1. **eFigure 1.** Selection of the Analytic Cohort **eFigure 2.** Life Space Assessment Used in the MrOS Study **eTable 1.** Association of Baseline Life Space and Change in Life Space With Incident Parkinson’s Disease (Ascertained by Physician Diagnosis or Physician Diagnosis Plus Dopaminergic Medication Use) Over 7 Years **eTable 2.** Association of Baseline Life Space and Change in Life Space With Incident Alzheimer’s Disease and Parkinson’s Disease Over 7 Years, Excluding 87 Individuals With Baseline Restricted Life Space **eTable 3.** Association of Baseline Life Space and Change in Life Space With Incident Alzheimer’s Disease and Parkinson’s Disease Over 7 Years, Excluding 89 Individuals With Baseline Cognitive Impairment

Supplement 2.
Data Sharing Statement
