# Supplementary material for: Constricting Life Space and Likelihood of Neurodegenerative Disease in Community-Dwelling Older Men
Source: JAMA Netw Open. 2023 Nov 9;6(11):e2342670. doi: 10.1001/jamanetworkopen.2023.42670 (PMC10636634; doi:10.1001/jamanetworkopen.2023.42670)
Supplement: Supplement 1. — eFigure 1. Selection of the Analytic Cohort eFigure 2. Life Space Assessment Used in the MrOS Study eTable 1. Association of Baseline Life Space and Change in Life Space With Incident Parkinson’s Disease (Ascertained by Physician Diagnosis or Physician Diagnosis Plus Dopaminergic Medication Use) Over 7 Years eTable 2. Association of Baseline Life Space and Change in Life Space With Incident Alzheimer’s Disease and Parkinson’s Disease Over 7 Years, Excluding 87 Individuals With Baseline Restricted Life Space eTable 3. Association of Baseline Life Space and Change in Life Space With Incident Alzheimer’s Disease and Parkinson’s Disease Over 7 Years, Excluding 89 Individuals With Baseline Cognitive Impairment [file jamanetwopen-e2342670-s001.pdf]

Supplemental Online Content

Bock MA, Hoang T, Cawthon P, et al. Constricting life space and likelihood of neurodegenerative disease in community-dwelling older men. *JAMA Netw Open*. 2023;6(11):e2342670. doi:10.1001/jamanetworkopen.2023.42670

eFigure 1. Selection of the Analytic Cohort
eFigure 2. Life Space Assessment Used in the MrOS Study
eTable 1. Association of Baseline Life Space and Change in Life Space With Incident Parkinson's Disease (Ascertained by Physician Diagnosis or Physician Diagnosis Plus Dopaminergic Medication Use) Over 7 Years
eTable 2. Association of Baseline Life Space and Change in Life Space With Incident Alzheimer's Disease and Parkinson's Disease Over 7 Years, Excluding 87 Individuals With Baseline Restricted Life Space
eTable 3. Association of Baseline Life Space and Change in Life Space With Incident Alzheimer's Disease and Parkinson's Disease Over 7 Years, Excluding 89 Individuals With Baseline Cognitive Impairment

This supplemental material has been provided by the authors to give readers additional information about their work.

© 2023 Bock MA et al. *JAMA Network Open.*

eFigure 1: Selection of the Analytic Cohort

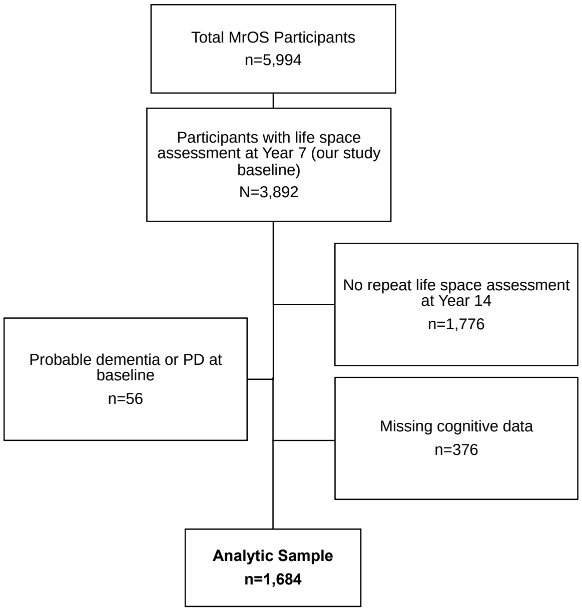

© 2023 Bock MA et al. *JAMA Network Open.*

eFigure 2: Life Space Assessment used in the MrOS study

Draft

Life Space

Office Use Only-
MrOS ID# | Acrostic | Staff ID# LSSTAFF

Was the Life Space Questionnaire administered? ○ Yes ○ No LSLSQ

These questions refer to your activities within the PAST MONTH (prior to today):

1. During the past four weeks, have you been to other rooms of your home besides the room where you sleep?
   - 0 ○ No → **GO TO QUESTION #2**
   - 1 ○ Yes → **How often did you get there?** LS1FQ
     1 ○ Less than 1/week 2 ○ 1-3 times/week 3 ○ 4-6 times/week 4 ○ Daily
     Did you use aids or equipment? Did you need help from another person? LS1IND
     2 ○ Personal Assistance 1 ○ Equipment Only 0 ○ No
   - LS1YN

2. During the past four weeks, have you been to an area outside your home such as your porch, deck, or patio, hallway (of an apartment building) or garage, in your own yard or driveway?
   - 0 ○ No → **GO TO QUESTION #3**
   - 1 ○ Yes → **How often did you get there?** LS2FQ
     1 ○ Less than 1/week 2 ○ 1-3 times/week 3 ○ 4-6 times/week 4 ○ Daily
     Did you use aids or equipment? Did you need help from another person? LS2IND
     2 ○ Personal Assistance 1 ○ Equipment Only 0 ○ No
   - LS2YN

3. During the past four weeks, have you been to places in your neighborhood, other than your own yard or apartment building?
   - 0 ○ No → **GO TO QUESTION #4**
   - 1 ○ Yes → **How often did you get there?** LS3FQ
     1 ○ Less than 1/week 2 ○ 1-3 times/week 3 ○ 4-6 times/week 4 ○ Daily
     Did you use aids or equipment? Did you need help from another person? LS3IND
     2 ○ Personal Assistance 1 ○ Equipment Only 0 ○ No
   - LS3YN

4. During the past four weeks, have you been to places outside your neighborhood, but within your town?
   - 0 ○ No → **GO TO QUESTION #5**
   - 1 ○ Yes → **How often did you get there?** LS4FQ
     1 ○ Less than 1/week 2 ○ 1-3 times/week 3 ○ 4-6 times/week 4 ○ Daily
     Did you use aids or equipment? Did you need help from another person? LS4IND
     2 ○ Personal Assistance 1 ○ Equipment Only 0 ○ No
   - LS4YN

5. During the past four weeks, have you been to places outside your town?
   - 0 ○ No → **GO TO TENG MINI-MENTAL**
   - 1 ○ Yes → **How often did you get there?** LS5FQ
     1 ○ Less than 1/week 2 ○ 1-3 times/week 3 ○ 4-6 times/week 4 ○ Daily
     Did you use aids or equipment? Did you need help from another person? LS5IND
     2 ○ Personal Assistance 1 ○ Equipment Only 0 ○ No
   - LS5YN

LSM LSE LSC
LSI LSII
LSID LSIII
PAGE 5

Version 1.0 01.19.2007 VISIT 3
MrOSIELifeSpace

Mr.OS

Draft

© 2023 Bock MA et al. *JAMA Network Open.*

eTable 1: Association of Baseline Life Space and Change in Life Space with Incident Parkinson's Disease (ascertained by physician diagnosis or physician diagnosis plus dopaminergic medication use) over 7 years

	Odds Ratio (95% CI)			
	Incident Parkinson's Disease (Physician Diagnosis) N=23		Incident Parkinson's Disease (Physician Diagnosis and Dopaminergic Medication use) N=17	
**Life Space Tertiles**	Model 1[1]	Model 2[2]	Model 1[1]	Model 2[2]
Least Restricted	Ref	Ref	Ref	Ref
Moderately Restricted	1.36 (0.48, 3.86)	1.23 (0.43, 3.51)	1.33 (0.45, 3.99)	0.96 (0.31, 3.47)
Most restricted	1.39 (0.53, 3.62)	1.25 (0.42, 3.72)	1.17 (0.34, 4.01)	1.06 (0.29, 3.92)
P-value[3]	0.49	0.71	0.60	0.96
**Life Space Change**				
1 SD Change in Life Space Score[4]	1.63 (1.11, 2.41)	1.48 (0.97, 2.25)	1.78 (1.14, 2.78)	1.02 (0.97, 1.04)
P-value	0.010	0.072	0.010	0.106

[1]Model 1 is unadjusted.
[2]Model 2 is adjusted for age, clinic site, race, education, living alone, diabetes, stroke, hypertension, depression, gait speed, and physical activity (PASE score).
[3]P-value is for the trend across life space tertiles.
[4]1 SD change in life space is 21.9 points.

© 2023 Bock MA et al. *JAMA Network Open.*

eTable 2: Association of Baseline Life Space and Change in Life Space with Incident Alzheimer's Disease and Parkinson's Disease over 7 years, Excluding 87 Individuals with Baseline Restricted Life Space

	**Odds Ratio (95% CI)**			
	**Incident Dementia N=80**		**Incident Parkinson's Disease N=23**	
**Life Space Tertiles**	Model 1[1]	Model 2[2]	Model 1[1]	Model 2[2]
Least Restricted	Ref	Ref	Ref	Ref
Moderately Restricted	0.54 (0.27, 1.09)	0.52 (0.22, 1.23)	1.36 (0.48, 3.86)	1.23 (0.43, 3.51)
Most restricted	1.39 (0.83, 2.31)	1.31 (0.76, 2.27)	1.39 (0.53, 3.62)	1.25 (0.42, 3.72)
P-value[3]	0.21	0.34	0.49	0.71
**Life Space Change**				
1 SD Decrease in Life Space Score[4]	1.58 (1.27, 1.98)	1.53 (1.20, 1.986)	1.74 (1.15, 2.62)	1.66 (1.05, 2.64)
P-value	<0.001	0.001	0.009	0.031

[1]Model 1 is unadjusted.
[2]Model 2 is adjusted for age, clinic site, race, education, living alone, diabetes, stroke, hypertension, depression, gait speed, and physical activity (PASE score).
[3]P-value is for the trend across life space tertiles.
[4]1 SD change in life space is 21.9 points.

© 2023 Bock MA et al. *JAMA Network Open.*

eTable 3: Association of Baseline Life Space and Change in Life Space with Incident Alzheimer's Disease and Parkinson's Disease over 7 years, Excluding 89 Individuals with Baseline Cognitive Impairment

	**Odds Ratio (95% CI) N=1,595**			
	**Incident Dementia N=74**		**Incident Parkinson's Disease N=21**	
**Life Space Tertiles**	Model 1[1]	Model 2[2]	Model 1[1]	Model 2[2]
Least Restricted	Ref	Ref	Ref	Ref
Moderately Restricted	0.57 (0.28, 1.17)	0.56 (0.27, 1.16)	1.11 (0.39, 3.14)	0.96 (0.31, 2.98)
Most restricted	1.26 (0.75, 2.10)	1.24 (0.71, 2.16)	1.41 (0.50, 3.99)	1.34 (0.45, 4.00)
P-value[3]	0.38	0.57	0.84	0.98
**Life Space Change**				
1 SD Decrease in Life Space Score[4]	1.66 (1.33, 2.07)	1.62 (1.27, 2.08)	1.63 (1.09, 2.44)	1.43 (0.93, 2.23)
P-value	<0.001	<0.001	0.018	0.11

[1]Model 1 is unadjusted.
[2]Model 2 is adjusted for age, clinic site, race, education, living alone, diabetes, stroke, hypertension, depression, gait speed, and physical activity (PASE score).
[3]P-value is for the trend across life space tertiles.
[4]1 SD change in life space is 21.9 points.

© 2023 Bock MA et al. *JAMA Network Open.*
