# Supplementary material for: Constricting Life Space and Likelihood of Neurodegenerative Disease in Community-Dwelling Older Men
Source: JAMA Netw Open. 2023 Nov 9;6(11):e2342670. doi: 10.1001/jamanetworkopen.2023.42670 (PMC10636634; doi:10.1001/jamanetworkopen.2023.42670)
Supplement: Supplement 2. — Data Sharing Statement [file jamanetwopen-e2342670-s002.pdf]

Data Sharing Statement

Bock. Constricting Life Space and Likelihood of Neurodegenerative Disease in Community-Dwelling Older Men. *JAMA Netw Open*. Published November 10, 2023.
doi:10.1001/jamanetworkopen.2023.42670

Data
Data available: Yes
Data types: Deidentified participant data
How to access data: Data from MrOS are available at mrosonline.ucsf.edu. The analysis dataset for this specific manuscript is available from the corresponding author upon request.
When available: With publication

Supporting Documents
Document types: Statistical/analytic code
How to access documents: The analysis code for this specific manuscript is available from the corresponding author upon request.
When available: With publication

Additional Information
Who can access the data: The analysis code for this specific manuscript is available from the corresponding author upon request.
Types of analyses: The analysis code for this specific manuscript is available from the corresponding author upon request.
Mechanisms of data availability: The analysis code for this specific manuscript is available from the corresponding author upon request with investigator support.
